# The Role of Colorectal Cancer Stem Cells in Metastatic Disease and Therapeutic Response

**DOI:** 10.3390/cancers3010319

**Published:** 2011-01-14

**Authors:** Eric C. Anderson, Crystal Hessman, Trevor G. Levin, Marcus M. Monroe, Melissa H. Wong

**Affiliations:** 1 Division of Hematology/Medical Oncology, Knight Cancer Institute, Oregon Health and Science University, 3181 SW Sam Jackson Park Rd, Portland, OR 97239, USA; E-Mail: andeeric@ohsu.edu; 2 Department of Surgery, Oregon Health and Science University, 3181 SW Sam Jackson Park Rd, Portland, OR 97239, USA; E-Mail: hessmanc@ohsu.edu; 3 Department of Cell and Developmental Biology, Knight Cancer Institute, Oregon Stem Cell Center, Oregon Health and Science University, 3181 SW Sam Jackson Park Rd, Portland, OR 97239, USA; E-Mail: levint@ohsu.edu; 4 Department of Otolaryngology—Head and Neck Surgery, Oregon Health and Science University, 3181 SW Sam Jackson Park Rd, Portland, OR 97239, USA; E-Mail: monroem@ohsu.edu; 5 Department of Dermatology, Department of Cell and Developmental Biology, Knight Cancer Institute, Oregon Stem Cell Center, Oregon Health and Science University, 3181 SW Sam Jackson Park Rd, Portland, OR 97239, USA

**Keywords:** colon cancer, metastatic, cancer stem cell

## Abstract

Colorectal cancer is the third-leading cause of cancer related mortality in the United States. The intricate molecular mechanisms involved in the regenerative process of the normal intestine and the identity of putative somatic intestinal stem cells have become clear. In parallel with this, experiment evidence has emerged supporting the century old hypothesis that solid tumor initiation, progression, chemoresistance and recurrence is the result of a small population of cancer cells with self-renewal and pluripotency capabilities. These “cancer stem cells” (CSCs) present a unique opportunity to better understand the biology of solid tumors in general, as well as targets for future therapeutics. In this review, we will summarize the current understanding of intestinal stem cell biology and translate it to colorectal CSCs to provide a basis for understanding chemoresistance, cancer recurrence and metastasis. A more complete understanding of the biology of colorectal CSCs will translate into the development of better chemotherapeutic and biological agents for the treatment of colorectal cancer.

## 1. Introduction

Colorectal cancer (CRC) is a leading cause of morbidity and mortality in the United States and worldwide. Although the underlying molecular events leading to the development of primary colorectal cancer are well understood, and advances in early detection have led to an overall decrease in the number of deaths, advanced and metastatic CRC is rarely curable. Over the past 15 years, evidence has emerged to suggest that cancers, including CRC, can be considered a stem cell disease. The cancer stem cell (CSC) theory posits that both primary and metastatic tumors develop from a small population of cancer cells possessing the characteristics of self-renewal and pluripotency and are responsible for initiation and maintenance of tumors. Additionally these CSCs can give rise to a wide variety of more “differentiated” cancer cells which comprise the bulk of the tumor and provide the basis of tumor heterogeneity. While the source of colorectal cancer stem cells remains to be completely elucidated, it is clear that because these cells behave in a manner similar to endogenous stem cells, a better understanding of the somatic intestinal stem cell and its niche will further our knowledge of the function—and dysfunction—of CSCs in the colon. In this review, we describe the role of CSCs in CRC with a focus on their role in metastatic disease. We illustrate that a basic understanding of the normal intestinal structure, function and stem cell niche lends insight into the initiation and progression of CRC. We then integrate the CSC theory and the role of CSCs in this process and extend it to metastatic spread of disease. Finally, we discuss therapeutic implications for the existence of CSCs.

## 2. Intestinal Structure and the Stem Cell Niche

**Figure 1.**
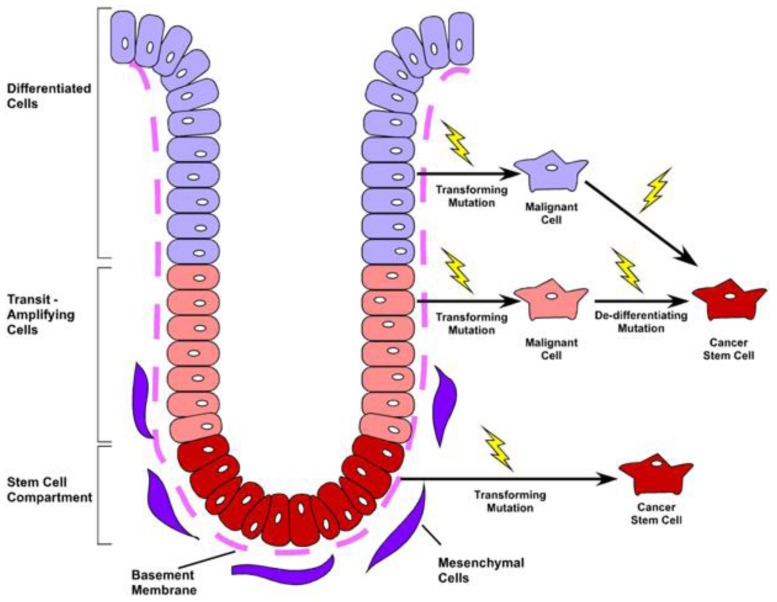
Diagram of the human colonic crypt structure. (Left) The stem cell compartment resides at the base of the crypt. Rapidly dividing transit-amplifying (TA) cells arise from this population and differentiate into the functional cells of the colon. (Right) The source of the colon CSC remains controversial. A single transforming mutation in a somatic intestinal stem cell could give rise to a CSC, while two mutations (one transforming and one de-differentiating) would be required to change a TA or differentiated colonic cell into a CSC.

The primary function of the gastrointestinal tract is to facilitate nutrient absorption and act as a barrier to the external environment. As such, the colonic lining has optimally evolved to accommodate both functions, by maximizing absorptive surface area and maintaining continual renewal of a barrier-tight sheet of epithelium. To accommodate these diverse functions, the colonic epithelium is organized as a contiguous layer of columnar epithelia arranged along the radial axis into distinct cryptlike structures. Within the base of the crypts, the intestinal stem cell provides continual renewal of the diverse epithelial subtypes. Four differentiated cell lineages reside within the colonic crypt and surface cuff epithelium: Colonocytes, the primary absorptive cell; goblet cells, the mucin secreting cell; enteroendocrine cells, the hormone-producing population; and in the cecum—and very rarely in the remainder of the colon—anti-microbial secreting Paneth cells [1]. Differentiated cells rapidly migrate up the intestinal glands and die or are sloughed into the lumen within 4–8 days [2]. These cells are continually repopulated by a long-lived, intestinal epithelial stem cell that resides in the base of the crypt (Figure 1). The stem cell is capable of self-renewal and also gives rise to the transit-amplifying (TA) cell population located near the lower portion of the crypt, which functions to rapidly expand epithelial renewal and initiate lineage differentiation. The resulting differentiated progeny migrate upward along the colonic crypt to the crypt opening. These intricate details and cellular relationships were first elucidated in the small intestine using BrdU and ^3^H-thymidine label retention studies along with electron microscopy [3-8]. Interestingly, although the location of intestinal progenitor cells has been known for some time, specific markers for these cells have only recently been characterized [9].

Insights into the identification of the intestinal stem cell have mainly focused on the small intestine rather than the colon. However, in both regions, the leucine-rich repeat-containing G-protein coupled receptor 5 (Lgr5) protein is expressed in a crypt-base, progenitor population capable of giving rise to all of the differentiated lineages within the intestine [10]. Further, this population has been shown to initiate intestinal organoid growth in a three-dimensional culture system when isolated from the mouse small intestine [11]. Culture conditions for both human tissues and mouse colonic cells are currently being investigated by a number of laboratories. While not all identified stem cell populations have been validated by both lineage tracing and *in vitro* assays, a number of other protein markers for the epithelial stem cell/progenitor cells have been identified. These include: BMI1 polycomb ring finger oncogene (Bmi-1) [12], Musashi-1 (Msi-1) [13], DCAMKL-1 [14], CD133 [15] and Activated Leukocyte Adhesion Molecule (ALCAM/CD166) which marks a broader stem cell region as a niche marker [16]. While the epithelial function for many of these proteins has yet to be elucidated, continued understanding of the populations that express them is certain to shed important insight into epithelial homeostasis, regeneration, and disease.

Currently, it is unclear if a hierarchical lineage relationship exists among the various progenitor cells of the intestine. It has been proposed that the Wnt-responsive gene Lgr5 exclusively marks actively dividing intestinal stem cells [9]. It is possible that a more dormant or quiescent population of stem cells is at the apex of the stem cell hierarchy and gives rise to the rapidly cycling Lgr5 progenitors in a similar fashion as the well-described hematopoietic and neuronal stem cell hierarchies. This type of relationship may help explain how the intestine regenerates after radiation exposure and chemotherapy, which target actively cycling cells (likely Lgr5-expressing populations) [17]. Solid tumors which develop resistance to these therapies may use a similar mechanism, in which a subset of cells capable of repopulating a tumor is in a dormant (protected) state during dosing of cytotoxic therapeutics. A progenitor cell hierarchy may also exist among the TA population where lineage restriction is initiated, resulting in generation of specific cell types [18]. Interestingly, dysregulation of these progenitor pools may be reflected in cancers where single cell types dominate the tumor, such as mucinous adenocarcinoma. A better understanding of differences between normal intestinal progenitors and their progeny will lead to greater insight into the various initiating cells within a cancer and has great potential to lead to novel therapeutic approaches for eradicating disease.

## 3. Colorectal Cancer and Metastatic Disease

Colorectal cancer (CRC) will account for approximately 150,000 new cases and 56,000 deaths in the United States this year, making it the third most commonly diagnosed cancer, as well as the third-leading cause of cancer related mortality [19]. The incidence of CRC has declined over the last two decades with the advent and implementation of routine screening colonoscopy, which allows for early detection and removal of adenomatous polyps before they progress to invasive cancer. Early detection and treatment is the key to better survival. Patients diagnosed with early stage CRC have a five year survival rate of greater than 90% compared to 11% for those diagnosed with locally advanced or metastatic disease. Furthermore, patients with metastatic CRC have a median survival of only two years despite multiple available treatment modalities, including surgical resection, chemoradiation, monoclonal antibodies to tumor growth factors, and liver-directed therapies for metastatic disease. Unfortunately, only a small subset of metastases are sensitive to these therapies and fewer still are cured, highlighting our lack of knowledge regarding the biological underpinnings of this most deadly phase of CRC.

A major challenge in treating metastatic CRC is the inability to predict tumor behavior and response to therapy *a priori*. In part, this is due to the complexity of molecular mutations that evolve within each individual cancer. The early pathway to CRC tumorigenesis has been well elucidated by Vogelstein and colleagues. Tumorigenesis is initiated when a single colorectal epithelial cell acquires a mutation in the tumor suppressor APC gene that controls the Wnt/β-catenin signaling pathway [20]. Mutations in the KRAS and BRAF genes enable growth into a clinically significant adenoma with a diameter >1 cm. Additional mutations in TGF-β, PIK3CA, and TP53 further drive clonal expansion and transformation from a benign adenoma to a carcinoma that now has the potential for invasion and metastasis. These mutations that cause an adenoma to transform into an advanced carcinoma occur over a long period of time, 15–20 years on average. However, cells within a carcinoma quickly acquire the potential to metastasize, as the average interval to liver metastases is approximately two years following diagnosis of an advanced carcinoma [20]. Despite an understanding of the mutations that give rise to a primary colorectal tumor, the molecular basis for the development of metastatic CRC remains largely unknown and clearly differs from that of primary tumorigenesis. The unique signatures displayed in metastatic CRC impart different functional behaviors and, interestingly, are also exemplified in a form of CRC seen within the young adult population (<50 years of age). In this younger population, the disease is much more aggressive with a shorter time to metastasis. Because screening is not routinely recommended, the incidence of CRC within the young adult population is actually increasing by 2% per year [19]. These two aggressive forms of CRC clearly exemplify the lack of understanding of the basic tumor biology driving this disease.

**Figure 2.**
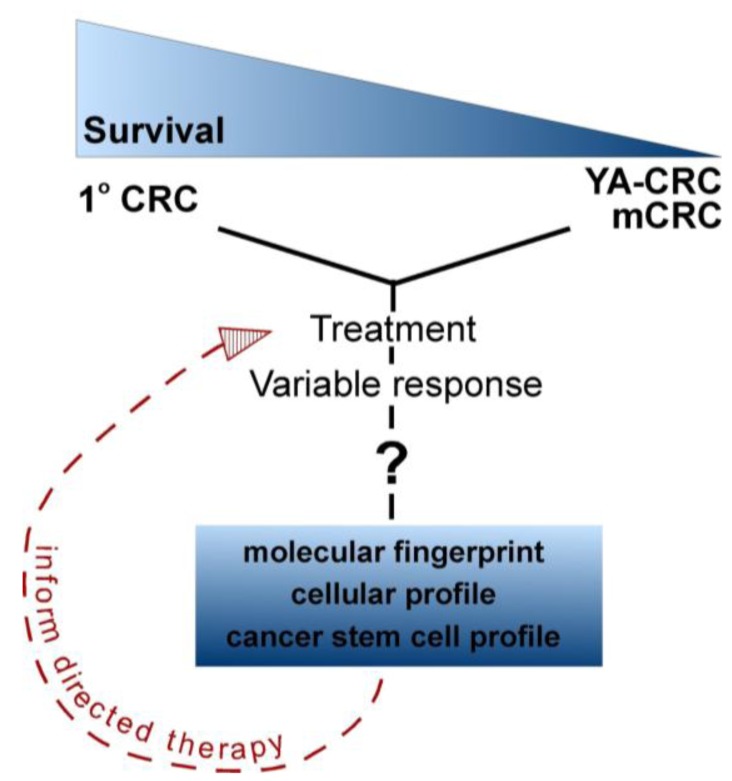
Colorectal cancer (CRC) has better survival odds than metastatic CRC (mCRC) or young-adult CRC (YA-CRC). The difference in disease response to the current state-of-the-art treatment reflects a gradient of disease with the early staged primary CRC (1°CRC) responding more favorably than late stage CRC, YA-CRC or mCRC. The variability in treatment response is likely dependent upon differences in molecular and cellular characteristics among the disease spectrum. The current challenge is to understand these differences to inform targeted therapy with the ultimate goal of cancer eradication.

It is not surprising that there are few effective targeted therapies for aggressive metastatic CRC. With the exception of K-ras mutations with anti-EGFR therapy and 5-fluorouracil treatment in microsatellite unstable tumors [21,22], the response of any individual tumor to a specific therapy must be determined empirically. New, potentially more effective therapies are evaluated only after traditional treatments fail. This also highlights the fact that the biology of primary and metastatic tumors differs in clinically important ways. This is not surprising, as metastatic tumor cells must evolve to escape the primary tumor niche, migrate and establish a new niche in a potentially hostile cellular environment. Whether these differences are due to molecular differences as a result of the accumulation of additional genetic mutations or a change in the cellular profile of the tumor (through epigenetic changes or post-translational regulation of tumor cells) remains to be determined (Figure 2). Therefore, a better understanding of tumor biology will provide valuable clues to therapeutic resistance as well as offer new targets for the development of novel chemotherapeutic and biological agents for the treatment of advanced and metastatic CRC.

There is a growing—although somewhat controversial—body of evidence suggesting that heterogeneous tumors harbor a specialized population of tumor-initiating cells that have been compared to endogenous stem cells. While these tumor-initiating cells may or may not truly be considered stem cells, it is clear that this specialized sub-population of tumor cells is able to recapitulate the heterogeneous tumor for all solid tumors examined to date, including CRC. As with somatic stem cells, these CSCs possess the ability to initiate and sustain tumor growth and have been shown to be resistant to damage and death after exposure to standard chemotherapeutic agents [23].

Given this new understanding of the mechanisms of tumor initiation and maintenance through the CSC, it is clear that a better understanding of the somatic stem cell and its niche will provide insight into the development of CRC in both its primary and metastatic environments.

## 4. The Cancer Stem Cell Theory

It has been long recognized that tumors are composed of a heterogeneous population of cells with various levels of cellular differentiation and morphologic features. At the same time, most tumors are believed to be monoclonal in origin [24,25], supporting the notion that the originating tumor must be capable of giving rise to various cell types that make up the tumor. Interestingly, for several decades, selection of mutant subpopulations derived from a common progenitor (clonal evolution), as well as microenvironmental influences, have been the predominant explanations for how a complex and heterogeneous tumor develops from a single cell. In addition, these selective pressures have been thought to provide the driving force for tumor growth and progression [26].

**Figure 3.**
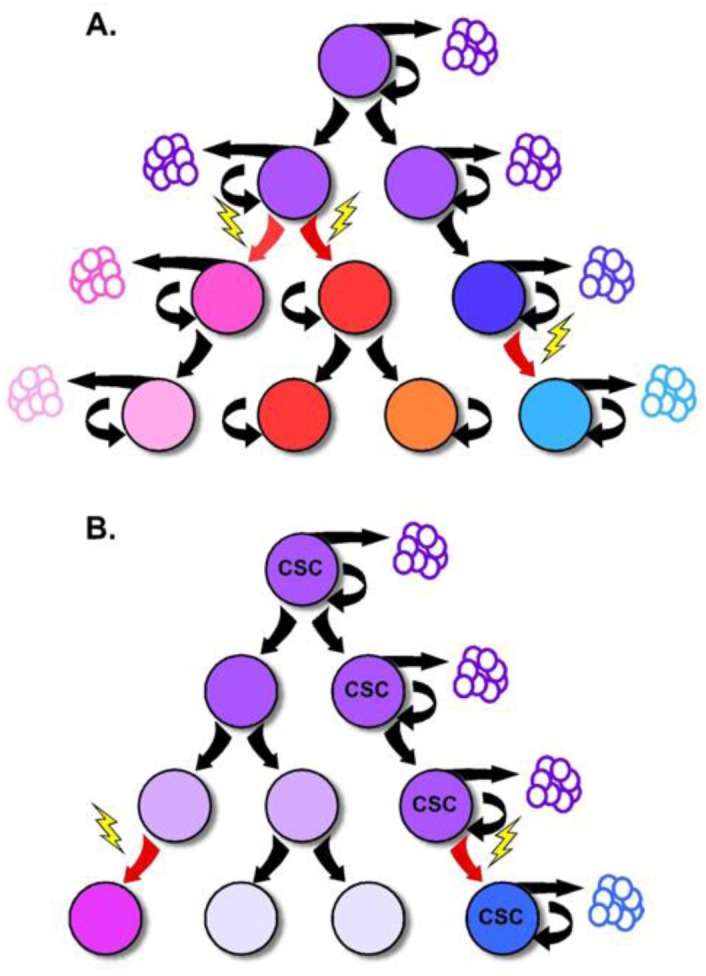
(**A**) Classical hierarchical model of tumorigenesis where any tumor cell has the potential and capacity to recapitulate the tumor, thus giving rise to tumor heterogeneity. (**B)** In the cancer stem cell (CSC) model of tumorigenesis, only CSCs have the potential to recapitulate the tumor. All other tumor cells are “differentiated.” Tumor heterogeneity arises as the result of mutations in the CSC and differentiation of its progeny.

Portions of this model have recently been challenged by increasing evidence that tumor growth and progression are supported by a small population of tumor cells with stem-like properties, and the reinvigoration of the CSC theory. While most normal tissues are supported by a small population of slowly cycling and self-renewing stem cells, the CSC theory proposes the existence of a similar tumor cell hierarchy with a CSC residing at the apex [27]. In this model, the self-renewing CSC divides to give rise to tumor cell subpopulations with more limited replicative ability that generally comprise the bulk of the tumor. Because of the difference in replicative capacity, the tumorigenic supporting abilities are thought to be exclusive to the CSC, while tumor growth and expansion is attributed to the rapidly dividing progeny. This critical point is the departure from previous models of tumorigenesis which support the notion that each tumor cell should be capable of tumor formation [28] (Figure 3).

While increasing evidence supports the existence of the CSC, the origins of this cell remain uncertain. Genetic or epigenetic changes may render a normal tissue stem cell cancerous, or may confer stem-like abilities on a progenitor or differentiated cell [29]. Because of this uncertainty, the terms “cancer-initiating cell” or “tumor-initiating cell” are often used interchangeably with “cancer stem cell.” The true definition of a CSC, however, is based upon its function—namely the capacity for self-renewal and the ability to give rise to the heterogeneous lineages of cancer cells that comprise a tumor [28].

The CSC theory of tumorigenesis, while receiving a great deal of attention recently, is based on concepts that have existed for over 150 years. As early as 1855, Rudolph Virchow proposed that tumors develop from residual embryonic nests (reviewed in [29]). Over the last century, this idea has been revisited multiple times. In the 1960s, evidence supporting the notion that not all tumor cells have an equal capacity for tumorigenesis was highlighted in quantitative tumor autotransplantation assays. In this study, tumor cell suspensions derived from patients with disseminated malignancy were injected subcutaneously into patients' own thighs. Based upon the high number of cells required for tumor growth, the authors speculated that the entire tumor cell population might be derived from a single CSC [29,30].

CSCs were first identified from the blood of patients with acute myelogenous leukemia (AML), by John Dick and colleagues in the 1990s [31,32]. Using xenotransplantation assays in NOD/SCID mice, they showed that tumorigenic potential resided with only a small subset of leukemic cells, characterized by high CD34 and low CD38 cell surface expression. Furthermore, when this population of leukemic cells was transplanted into immunocompromised mice, they developed AML that was phenotypically similar to the subtype of AML present in the patient from which the cells were originally derived.

Several years later, Clarke and colleagues were the first to prospectively identify CSCs in a solid malignancy [33]. Using similar xenotransplantation assays, they identified a breast cancer cell population characterized by high CD44 and low CD24 expression that recapitulated the original tumor phenotype and developed from as few as 100 transplanted cells. Conversely, transplantation of tens of thousands of the alternate cellular phenotypes did not give rise to new tumors. Since that time, a multitude of studies have been published characterizing CSC populations across a wide variety of solid organ malignancies, including CNS, pancreatic, head and neck, and colorectal cancers [34-37].

The CSC theory, aside from the contribution to our understanding of tumor biology, has potential far-reaching clinical implications. Like their normal tissue counterparts, CSCs have been shown to display increased chemoresistance and radioresistance [23,38-42]. Traditional cancer therapies typically target the rapidly dividing tumor cell population and, as increasing evidence suggests, may preferentially spare the CSC component of the tumor [39,41]. This may explain the often-encountered clinical scenario in which a tumor has apparent complete volumetric tumor reduction followed by subsequent local recurrence. As such, the CSC theory suggests that not only will our therapeutic targets need to be re-envisioned with a focus on the CSC, but our methods for measuring therapeutic efficacy will need to be revised as well.

## 5. Stem Cell Hierarchy in Colorectal Tumors

Tumorigenesis within the colon follows an adenoma-carcinoma sequence first described in the early 1990s by Fearon and Vogelstein. The observation that colorectal tumors arise from a series of mutations that lead to the activation of oncogenes, inactivation of tumor suppressor genes and result in unregulated growth, has provided the framework for our understanding of tumor biology in the colon. While it is clear that mutations in multiple genes are required for malignant transformation, fewer changes are sufficient for benign tumor growth [43]. Additionally, the fact that stochastic acquisition of mutations within various combinations of signaling pathways can lead to cancer suggests that acquisition of CRC is an inevitable, temporally dependent event [20]. Incorporating this concept into the CSC model implicates these mutations to occur within the long-lived stem cell, leading to an accumulation of multiple mutations over time [18] (Figure 3). The mutated stem cell can, in turn, give rise to additional mutated stem and progenitor cells through symmetric and asymmetric division, seeding tumor growth with mutated, transformed and heterogeneous cells. In this fashion, the CSC is capable of nurturing its own microenvironmental niche, as the survival of its diverse population is selected by the surrounding tumor stromal cells. In support of this idea, Vermeulen and colleagues showed that establishment and maintenance of the CRC stem cell niche is dependent on the Wnt signaling pathway orchestrated by myofibroblasts, suggesting that microenvironmental cues are as critical to the molecular diversity of tumors as are mutations [44].

Metastatic spread of disease is also consistent within the CSC theory. Independent subclonal populations within the tumor are endowed with different functional properties, but only selected clones have the potential to metastasize to distant organs [27]. In this model, the metastatic cells might originate from a monoclonal expansion of the original clonal cell population. But over time, development of additional genetic mutations enable responsiveness to environmental signals and acquisition of metastatic properties; namely the ability to invade the surrounding region, intravasate through vasculature, evade the immune system and extravasate at a distant site [27]. In support of this acquired diversity, metastatic tumors have the potential to significantly diverge morphologically from the primary tumor. Recent evidence from gene-expression microarrays support the CSC model for metastases in epithelial tumors, including colon cancer [18].

While direct evidence for the origin of CSCs in human cancer is lacking, elegant mouse experiments by Clevers and colleagues demonstrated that ablation of the Apc gene in the Lgr5-expressing progenitor cell population was sufficient to drive development of intestinal adenomas. In contrast, when Apc was deleted in the more differentiated TA cell compartment, macroadenomas did not develop. Experiments from this well-studied intestinal tumor model system suggest that tumorigenesis is the result of malignant transformation specifically of a somatic tissue stem cell [45].

## 6. Identity of Colorectal Cancer Stem Cells

Certain barriers complicate the identification and isolation of CSCs within a tumor. Among these obstacles are the facts that stem cells are relatively scarce and lack a unique morphology that is easily distinguished from its progeny *in vivo* [46], and that CSCs are defined functionally by their ability to initiate tumorigenesis and, as such, can only be truly identified *post hoc*. Despite these hurdles, multiple studies have demonstrated that small, isolatable populations of human tumor cells exist that are capable of recapitulating the phenotype of the parental tumor when transplanted and grown in immunodeficient mice. To date, these cell populations have been isolated based on expression of cell surface markers and have been shown to comprise approximately 1% of the total number of cells within the cancer (Table 1).

An early study conducted by O'Brien *et al.* focused on validating CD133 as a colorectal CSC marker. In these experiments, CD133^+^ and CD133^−^ cells were isolated from both primary and metastatic human CRCs, and injected under the renal capsule of NOD/SCID mice. CD133^+^ cells gave rise to tumors while explanted CD133^-^ cells did not support tumor growth. Further, the regenerated CD133^+^ tumor cells could be serially transplanted and still retain the parental tumor morphology [54]. This observation has been recapitulated by other groups [55]. Furthermore, the CD133^+^ tumor cells showed exponential *in vitro* growth as tumor spheres, while maintaining the ability to generate new tumors when injected into immunodeficient mice. Upon withdrawal of growth factors, the cells within the tumor spheres gradually differentiated, resulting in loss of CD133 expression, and subsequent loss of their tumorigenic potential. Clarke's group used similar xenograft techniques to show that CD44^+^/CD166^+^/EpCAM^High^ cells isolated from human CRC could also establish a phenocopied tumor while no growth was observed with CD44^-^/CD166^-^/EpCAM ^Low^ cells. In addition to CD133, CD166, CD44 and EpCAM, a potential colon cancer stem cell marker is proposed to be the somatic intestinal stem/progenitor cell marker Lgr5 [56]. The importance of any one specific CSC marker identifying a “true” CRC stem cell population remain in flux, and several recent studies have questioned whether the CSC population remains static (e.g. expresses one specific marker, such as CD133, continuously throughout the course of disease), or whether this expression—and CSC function—is variable and potentially cyclic [34,57-60]. A recent and elegant examination of CD133 surface expression in glioblastoma multiforme highlighted this point by illustrating that the underlying PTEN signaling status represented a better correlation with CSC function than CD133 cell surface expression [59].

The question of whether these cell surface markers have functional relevance to the CSC population or whether they act simply as surrogate markers for CSCs remains unclear. Many of these proteins, such as CD133, have unknown function. Others, such as CD44 (hyaluronic acid receptor) and CD26 (dipeptidyl peptidase IV), have known functions; however, their functional relevance to tumorigenesis is uncertain and it is quite likely that these proteins have additional, currently unknown roles which may be relevant to cancer initiation or progression. As an example, CD166 is a member of the immunoglobulin super-family and is known to form homo-dimeric complexes as well as hetero-dimeric complexes with CD6 on lymphocytes to facilitate cell-cell interactions. Recent work in our laboratory has shown that CD166 marks the stem cell niche in the intestinal crypt in both mice and humans [16]. This suggests that CD166-expressing cells are important for the establishment and maintenance of the endogenous intestinal stem cell niche and, by extension, the CSC niche. Additionally, CD166 and other CSC marker proteins possessing cell-cell interactions may function to establish a pre-metastatic niche in target organs such as the liver, preparing a site to which migrating CSCs can home and establish metastatic deposits [16,61-64].

The role of CSCs in the establishment and maintenance of metastatic disease has been evaluated in several recent studies. Odoux and colleagues identified CD133^+^ and CD44^+^/CD166^+^/EpCAM^High^ cells in samples of metastatic CRC which maintained their CSC marker and histologic phenotypes in a limiting-dilution *in vitro* culture system as well as in *ex vivo* xenograft tumor models [65]. These results show that metastatic colorectal tumors possess similar CSC phenotypes and functionality as primary CRC tumors do. Further, CD133^+^ cells from the CRC cell line SW480 have enhanced migratory ability *in vitro* [60]. Analysis of metastatic CRC samples from peritoneal washings and comparison to the CRC tumor cell line HCT116 by Botchkina *et al.*, identified similar CD133^+^ and CD44^+^/CD166^+^/EpCAM^High^ cell populations with tumorigenic potential similar to prior studies [66,67]. These studies suggest that the biological basis of metastatic establishment is similar to that of the establishment of primary colorectal tumors. New data from Clarke and colleagues in *ex vivo* models of human breast cancer stem cells suggests that the CSCs responsible for metastatic formation are the same CSCs as those that develop primary tumors. Additionally, these CSCs escape the primary site of tumor implantation in a xenograft model before there is an obvious histologically invasive phenotype at the primary tumor site. These data suggest that some—although clearly not all—CSCs have obtained the invasive and migratory phenotype required for the establishment and maintenance of metastatic disease early in their development [67]. It remains to be determined whether this is a property of colorectal CSCs.

## 7. Clinical Implications of Cancer Stem Cells in Colorectal Cancer

While it is clear from available evidence that CSCs play an important role in CRC development and metastasis, the prognostic impact of tumor CSC content in any particular tumor remains unsettled. The extent to which CSC marker expression patterns can be used to predict survival or response to therapy is also unclear. While individual CSC markers (and combinations thereof) such as CD44, CD166 and CD133 [68-70] have been used to identify colorectal CSCs in specific patient populations, the prognostic and predictive utility of CSC markers remains uncertain, particularly in completely resected or widely metastatic disease [71-74]. Because the expression of these markers can be determined on virtually any type of tumor tissue (freshly isolated single-tumor cells, fresh-frozen tissue, archived formalin-fixed paraffin-embedded (FFPE) tissue, fine needle aspirates, or tumor cells isolated from peritoneal fluid or pleural effusions) using widely available technologies including flow cytometry, bright-field immunohistochemistry and multi-label immunofluorescence, the use of CSC markers and phenotype to predict clinical behavior such as metastatic potential and susceptibility to chemotherapy and radiation is an area of significant clinical importance. The ability to predict these behaviors will allow for more personalized and directed therapies based on tumor CSC phenotype.

Although surgical resection of metastatic disease is an option for some patients, the vast majority of cases of metastatic CRC are not amenable to curative surgical or radiation therapy, leaving chemotherapy and biologic therapy as the mainstays of treatment. While these treatments extend survival, they are not curative. The stem cell theory of tumorigenesis and metastasis states that a primary mechanism of treatment resistance in metastatic disease is the resistance of CSCs to traditional chemotherapy. As standard cytotoxic therapies target rapidly dividing tumor cells with the goal of maximum cytoreduction, the underlying tumor-maintaining cells (CSCs) divide less frequently and express drug efflux pumps similar to somatic stem cells, rendering them less susceptible to chemotherapy. The clinical manifestation of this biologic phenomenon is that, although many tumors initially respond well to chemotherapy resulting in radiographic complete remission of disease, more often the CSC remains at the site of disease, undamaged by chemotherapy, and able to initiate disease recurrence. Chemotherapy resistance of CSCs has been described in a variety of epithelial malignancies including breast, lung, head and neck, and pancreatic cancer [39,42,75,76]. Recent studies have shown similar data for CRC as well. Using EpCAM^+^/CD44^+^ colon cancer xenografts, Dylla and colleagues showed that this was the only tumor-initiating cell population remaining following treatment with the cytotoxic drugs irinotecan and cyclophosphamide, and that these cells express high levels of ALDH1, a gene implicated in chemoresistance and a marker of CSCs [41,77-79]. Chemotherapy resistant CRC cell lines HT-29/5FU-R and HT-29/OxR are enriched in CD44^+^/CD133^+^ CSC phenotypic cells [80]. Together, these studies suggest the importance of targeting both the bulk cancer cells and the tumor-initiating cell if any systemic anti-tumor therapy is to ultimately be successful.

**Figure 4.**
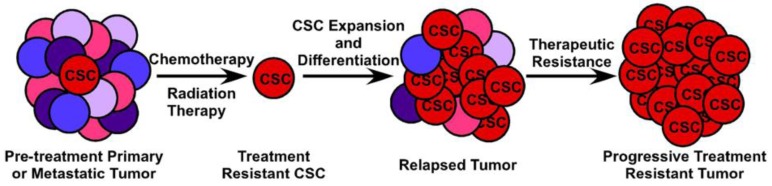
Clinical implications of the CSC model. Systemic chemotherapy and loco-regional radiation therapy affect the more differentiated tumor cells but not the CSC. Following therapy, the treatment-resistant CSC remains and is able to re-populate the tumor and give rise to additional treatment-resistant CSC progeny as well as chemotherapy-sensitive differentiated cells. Clinically, this is seen as disease relapse. Further treatment with standard cytotoxic and biologic therapies will result in increasing numbers of CSCs, which presents clinically as progressive, completely treatment-resistant disease.

The clinical implications of CSCs in metastatic CRC are manifold and quite significant. First, while the bulk of tumor cells will succumb to cytotoxic and biological therapy, remnant treatment resistant CSCs will remain, leading to disease recurrence most likely with decreased susceptibility to chemotherapy (Figure 4). Second, CSCs likely possess dysregulated signaling pathways such as the p53, WNT and Notch pathways, which are not targeted by current therapeutic agents. Targeting of the p53 pathway has failed to be fruitful under *in vivo* conditions and targeting of the WNT signaling pathway has thus far proven toxic (Reviewed in [81]). Finally, in order to completely eradicate a tumor and all of the CSCs which contribute to its survival, they must be targeted in a directed and specific manner. All of the markers currently used to identify CSCs *in vivo* are expressed on a variety of normal somatic cells, including somatic stem cells. Therapies targeted at any single CSC marker, such as monoclonal antibodies conjugated to cytotoxic compounds, is likely to also damage the normal tissue stem cell compartment, potentially leading to unacceptable toxicity. Because of the difficulty in prospectively identifying and maintaining tumor-initiating cells *in vitro*, identification of CSC-specific compounds has been slow and complicated. The use of breast cancer cells induced into an epithelial-mesenchymal transition (EMT) and enriched for CSCs in a high-throughput compound screen identified salinomycin as a CSC-targeting agent [82] and induces apoptosis in a variety of human hematologic cancer cell lines [83]. However, its efficacy against purified CSCs or other solid tumors has not been evaluated either *in vivo* or *in vitro*. A novel, immunotherapy approach to targeting tumor-initiating cells has been recently described by Herrmann and colleagues. Using MT110, a bi-specific antibody to EpCAM and human CD3 [84], this group was able to eliminate primary human colorectal tumors in a xenograft model, as well as xenografts generated from the HT29 CRC cell line by inducing tumor-specific T-cell cytotoxicity while avoiding apparent toxicity to the host animal [85]. Interestingly, this method eradicated both the CSC component of the tumor as well as the bulk tumor cell population. It is uncertain, however, whether the anti-EpCAM portion of the antibody would bind to normal EpCAM-expressing intestinal epithelium, inducing a similar cytotoxic response in normal colon epithelium and colonic stem cells. Giorgio Stassi and colleagues showed that CD133+ colorectal CSCs produced IL-4 which was able to protect these cells from chemotherapy-induced apoptosis. Blocking of the IL-4 activity with a neutralizing antibody or inhibitory IL-4 mutant subsequently sensitized these CSCs to 5-fluorouracil and oxaliplatin-induced death [86]. Further work from this same group shows that after treatment with zolendronic acid, CD133+ CRC CSCs can be effectively killed *in vitro* by γδ-T lymphocytes [87]. Clearly, while important discoveries are being made in the identification of CSC-targeting compounds, much work, particularly in Phase 0 and Phase I human studies, remains to be carried out.

## 8. Prospectus

The CSC hypothesis is revolutionizing the understanding of tumor initiation and progression, however much remains to be elucidated regarding the role of these specialized cells in metastasis and response to therapy, arguably the most clinically important aspects of tumor biology. While a number of studies have shown a correlation between the expression of CSC markers such as CD133, CD44, CD166 [88], Lgr5 [89] and Bmi1 [90] and survival, much less is known about the correlation of expression of these markers and the CSC phenotype in metastatic disease. More importantly, almost nothing is known about the functional relevance of these markers for tumor behavior. In a case-controlled study, Horst *et al.* examined CD133 expression in colonic tumors from patients with or without synchronous liver metastases and found increased expression of CD133 in the metastatic tumors compared to the localized tumors, but found no effect on proliferation, migration, or invasion when it was knocked down in cancer cell lines [91]. They concluded that while CD133 was highly prognostic for development of metastases, it had no functional relevance to the tumors. An alternative view holds that, although CD133 expression *per se* is not relevant to the metastatic phenotype, a pathway involving CD133 likely is important. As so little is known about the function of CD133, or many of the other CSC markers currently used, a better understanding of the functions and interactions of these proteins in cancer and normal somatic stem cells will be critical in furthering our understanding of the function and therapeutic targeting of the CSC.

Additional work is also needed to determine either a reliable surface identity of the colorectal cancer stem cell population, or—more likely, given the phenotypic and genetic variability between different tumors and over time in a single tumor—a panel of markers that precisely identifies the CSC. To date, most studies have evaluated one to at most four CSC markers in identifying a CSC population. This is largely due to technical limitations in the use of fluorescently labeled antibodies and the spectral limits of detection and fluorophore separation of most flow cytometers and microscopes. The use of new technologies such as quantum dot-antibody conjugates will allow for the simultaneous detection of increasing numbers of CSC markers and more precise CSC identification [92].

Currently, screening assays for the effectiveness of novel chemotherapeutic compounds largely rely on their *in vitro* cytotoxicity. The CSC model therefore has important implications and provides exciting new tools with respect to the design of new assays to test anticancer therapies. Three-dimensional tumorsphere culture systems can be generated from cancer cell lines or primary tumor cells enriched for CSC marker expression (and proven to be tumor-initiating cells in xenograft models) and used in high-throughput compound screens similar to current assays. Secondary screens can then be performed on promising compounds using orthotopic and heterotopic xenograft models of the sorted cell lines and tumors. One issue that must be addressed, however, is the need for a standardized methodology for identification and culturing of CSCs in order to allow clinically meaningful comparisons between different experimental compounds.

Because of the related features and functions of CSCs and normal somatic stem cells, it is clear that a significant limitation to designing compounds which target the CSC will be limiting their effects on the normal somatic stem cell. If somatic intestinal stem cells are damaged by drugs targeted to colorectal CSCs, it is likely that gastrointestinal toxicity would be unacceptably high and, unlike current chemotherapies which spare the stem cell population, may be fatal. Thus, while the use of normal intestinal stem cells to understand colorectal CSC biology is important, the identification of novel and unique CSC targets distinct from somatic stem cells is critical [93].

## 9. Conclusions

Increasing evidence supports the presence of a CSC or tumor initiating cell as the cause of tumor establishment, progression, relapse and metastasis. Identification of the origin of the CSC remains elusive in human CRC; however progress is being made in mouse models of intestinal cancer. The precise role of the CSC in these tumorigenic steps of CRC also remains unclear. Additionally, the interaction of colorectal CSCs with the cellular microenvironment, both at the site of tumor initiation and at sites of metastatic deposit, must be further investigated. This is particularly needed given the importance of the microenvironmental niche in the function and maintenance of somatic stem cells. Finally, in order to specifically target CSCs while sparing somatic intestinal stem cells, it will be critical to identify unique molecules and dysregulated pathways in the CSC population when compared to the somatic stem cell population. A better understanding of these aspects of somatic and CSC biology will be necessary in order to effectively target CSCs and ultimately develop cures for advanced and metastatic CRC.

## Figures and Tables

**Table 1.** Colorectal Cancer Stem Cell Markers.

**Marker Name**	**Function(s)**	**Ref.**
CD44	Hyaluronic Acid Receptor; Cell Adhesion (Osteopontin, collagens and MMPs)	[34,47,48]
CD133/Prominin1	Self-renewal	[49-52]
CD166/ALCAM	Cell Adhesion (Heterotypic/Homotypic)	[34,48]
ALDH1	Enzyme - Alcohol Metabolism	[53]
Lgr5	Wnt-target gene, function unknown	[48]
EpCAM/ESA	Homotypic Cell Adhesion	[34]
